# Advancing a cross-cultural understanding of teacher perceptions of school climate: A latent class analysis using 2018 TALIS data

**DOI:** 10.3389/fpsyg.2023.1129306

**Published:** 2023-03-09

**Authors:** Mingren Zhao, Rui Jin

**Affiliations:** Faculty of Education, Shenzhen University, Shenzhen, Guangdong, China

**Keywords:** cultural differences, school climate, latent class analysis, measurement invariance, TALIS 2018

## Abstract

In recent years, school climate has increasingly received research attention. Most studies have focused only on student perceptions of school climate, whereas little is known regarding teachers’ views, and cross-country comparisons are scarce. To advance cross-country understanding of teacher perceptions of school climate, this study used data from the 2018 Teaching and Learning International Study (TALIS) to explore latent classes of teacher perceptions and compared differences between American, Finnish, and Chinese teachers. Latent class analysis revealed that a four-class solution was the most appropriate for each teacher subsample: positive participation and teacher-student relation, positive teacher-student relation, moderate, and low participation for the U.S. and China datasets, while positive teacher-student relation, moderate, negative discipline, and low participation for the Finland dataset. However, measurement invariance across countries was violated. We further investigated the impact of predictors on latent classes of teacher perceptions of school climate. The results revealed varied patterns of cross-cultural differences across countries. Our findings implied that a more reliable and valid scale of teacher perceptions of school climate for cross-country comparison is needed. Tailored interventions are necessary as more than half of teachers perceived moderate and less desired school climate, and educators should consider cultural differences when drawing on experiences from other countries.

## Introduction

School climate comprises shared beliefs, values, and attitudes that shape interactions between students, teachers, and administrators (Mitchell et al., 2010; Gonzálvez et al., 2022). Extant research has suggested that school climate could not only have direct consequential effects on student success (Thapa et al., 2013; Wang and Degol, 2016; Izaguirre et al., 2023, p. 2023), but also significantly impact teacher outcomes, such as attrition (Djonko-Moore, 2016), efficacy (Meristo and Eisenschmidt, 2014; Hosford and O’Sullivan, 2016), job satisfaction (Zakariya, 2020), and burnout (Konold and Shukla, 2017; Capp et al., 2020; Yang et al., 2022). However, knowledge regarding teacher perceptions of school climate is limited, as most existing studies have been student-focused (Capp et al., 2022; Gonzálvez et al., 2022, p. 202; Wang and Degol, 2016).

Moreover, although students and teachers share objectively similar school environments, their perceptions vary significantly and even conflict due to the different roles and opportunities of teachers and students for observing school climate (Berkowitz et al., 2017; Maxwell et al., 2017; Capp et al., 2021). For instance, teachers, who control daily activities and tasks, reported more positive perceptions of climate than students (Ramsey et al., 2016; Molinari and Grazia, 2022). In addition, teachers were more concerned with classroom-level factors, such as the inconsistent implementation of rules, whereas students were more sensitive to issues at the school level, such as student mobility (Capp et al., 2021; Finch et al., 2023).

A more fundamental question is whether school climate can be simply dichotomized as positive or negative or a more complicated taxonomy is required. A supportive school climate serves not only as a safeguard for students (Berkowitz et al., 2017), but also a protective factor for teachers (Berkowitz et al., 2022). In contrast, teachers working in an unprotected environment may suffer a distressing effect. Perception of negative climate may be a reflection of teachers’ contributions to the school and could indicate that they had unpleasant experiences (Capp et al., 2020; Metrailer and Clark, 2022; Herman et al., 2023). Differences in how teachers feel about school climate imply that individual teachers may experience negative or positive school climate; however, there is likely to be a gradation of negative or positive climate (Capp et al., 2021). Exploring the diversity of teacher perceptions could provide alternative information regarding the climate and reveal areas that need improvement and intervention (Ramsey et al., 2016; Maxwell et al., 2017; Baumgarten et al., 2022).

This study used three domains of school climate (i.e., disciplinary climate, teacher-student relationships, and participation among stakeholders) as an initial point to investigate teacher perceptions of climate in different countries. Furthermore, this is one of the first studies to use the latent class model to analyze cross-country comparisons in teacher perceptions of school climate, showing how cultural and contextual factors shape the latent classes of teacher perceptions (Salle et al., 2015).

## Literature review

### Teacher-focused school climate

As previous literature has mainly concentrated on school climate as perceived by students, the primary definition, theoretical framework, and measurement are based on students’ views on climate and its impact on student outcomes, leading to the absence of teacher perspectives of school climate (Berkowitz et al., 2017; Molinari and Grazia, 2022, p. 202; Wang and Degol, 2016). To capture the unique components that make up the experiences of climate among teachers, Capp et al. (2020) proposed a conceptual and empirical model assuming that what teachers encounter as part of their regular tasks, interactions, and engagements has the power to alter how they feel about school climate. For instance, the authors listed two teacher characteristics that potentially influence teachers’ views on climate: school levels (i.e., elementary, middle, and high school) and teaching experience. Teachers in elementary schools feel better about school climate than teachers in middle and high schools because of an increase in student violence in middle and high schools compared to elementary schools. Novice teachers perceive less favorable climate than their experienced colleagues due to lower resource support or financial incentives (Koth et al., 2008; Milton et al., 2022).

However, due to data limitation, the conceptual model proposed by Capp et al. (2020) did not include a critical demographic variable: teacher gender. Empirical literature showed that female teachers perceived higher levels of climate than male teachers (Bevans et al., 2007; Shehbaz et al., 2022), as they displayed higher degrees of affinity and lower levels of conflict in their interactions with pupils than male teachers (Drugli, 2013). Moreover, a close teacher-student relationship could protect students from adverse effects, including peer exclusion and shyness (Roorda et al., 2011; Zee and Roorda, 2018; García-Rodríguez et al., 2022), which promotes a disciplinary climate. The present study empirically explored the differences in perceived school climate between female and male teachers and hypothesized that female teachers would report more positive perceptions of climate than male teachers.

### Cross-cultural comparisons of teacher perceptions of climate in three countries

The model proposed by Capp et al. (2020) and empirical studies (e.g., Capp et al., 2021) focusing on teachers’ perceptions of school climate were based on a single cultural context. Little is known about the role of cross-cultural factors in triggering the perception patterns of climate among teachers. Revealing cross-cultural differences is vital for designing culturally sensitive policies and interventions to encourage teacher participation in schools worldwide (Jahreie, 2022). Moreover, cross-cultural similarities shed light on the analogous and comparable aspects of the school settings and structural similarities, which could inform educators to transfer successful school-based practices from one nation to another (Berkowitz et al., 2017). Cross-cultural similarities also provide information about the similar and consistent parts of education systems that might provide consistency and connectedness for teachers, such as exchange teachers who are moving between countries.

To compare teacher perceptions of school climate in various cultural contexts, the present study used a sample from one European country (Finland), one North American country (the United States), and one Asian country (China). This study was rooted in the cultural-ecological model of school climate suggesting that teachers’ perceptions of climate are impacted by an interplay of individual, cultural, and environmental influences (Salle et al., 2015; Chen et al., 2019; Yang et al., 2021). This model clarifies cultural contexts and their contributions to individual perceptions of school climate in general and culturally specific ways, highlighting the necessity to examine how cultural factors impact perceptions of climate and explore cross-cultural comparisons.

These three countries are of particular interest due to their considerable contrasts in three dimensions of Hofstede’s (2005) cultural dimensions theory (Chen et al., 2019): power distance (the degree of inequality in society), individualism (the degree of individualism in society), and masculine (i.e., the degree a person is driven by material success rather than the relationships and quality of life). On a scale ranging from 0 to 100, American society expects low supervisor-subordinate relationships (scores 40 on power distance), extremely enjoys individual freedom (scores 91 on individualism), and moderately chases after earthly achievement (scores 62 on masculinity) (Hofstede et al., 2005). Finnish society extremely disputes unequal relationships (scores 33 on power distance), moderately values individual freedom (scores 63 on individualism), and extremely enjoys sound relationships and quality of life (scores 26 on masculinity) (Hofstede et al., 2005). Chinese people generally accept unequal relationships among people (scores 80 on power distance), highly value collectivism (scores 20 on individualism), and moderately pursue material success (scores 66 on masculinity) (Hofstede et al., 2005).

These cultural differences between the three countries have inevitably been reflected in their education systems, teaching practice, and stakeholder relationships (Barrenechea et al., 2022). For instance, education in China places a strong emphasis on the acquisition of knowledge, as well as the ways in which students organize and use the information they have gained in school (Pelgrum, 2001). Students in the Chinese school system are under a great deal of pressure to do well academically, and the system is often portrayed as being very competitive and stressful. Pupils in the U.S. are encouraged to critique, question, and invent new concepts or ideas, since American teachers are more focused on teaching students how to apply the information they obtain in the classroom to real-world situations (Halpern, 1998; Reilly and Reeves, 2022). Teachers in Finland prioritize fairness above academic achievement and attempt to prevent standardized examinations from being used as a tool for comparing pupils (Sahlberg, 2015).

Applied to perceptions of school climate, teachers’ cultural beliefs and attributions are highly impacted by the diverse traditional cultures and education systems (Abacioglu et al., 2022). This is important to cross-national comparisons of climate, as teachers spread and pass on the dominant cultural values, norms, and practices across classrooms, schools, and education systems through their teaching practices and interactions with students, peers, and principals (Kang, 2022). By including teachers from the three countries, the present study provided an opportunity to explore cross-national differences, which could increase our scientific knowledge of the relationship between school ecological factors and teacher perceptions of school climate and carry implications for designing interventions of school climate in different countries.

### Latent class analysis

Latent class analysis (LCA) has been extensively used to identify latent classes of individuals in the education field (Lin et al., 2018). LCA can extract exclusive and exhaustive subgroups with homogeneous characteristics (i.e., share a common pattern of responses) from a heterogeneous population (Goodman, 1974; Lanza et al., 2007). It serves the same primary objective as traditional cluster analysis (e.g., hierarchical clustering), which is to identify classes of observations with homogenous response patterns to a set of items (Ma, 2021). However, LCA is a data-driven approach that classifies individuals into derived classes, which are empirically generated (Rosato and Baer, 2012); thus, it could provide more robust and reliable classification results (DiStefano and Kamphaus, 2006). Moreover, multigroup LCA could be employed to assess differences in latent class structure across samples (Fan et al., 2019), which helps explore the similarities and differences of latent class of teacher perceptions of school climate across three countries in our study.

### Present study

Under the guidance of the cultural-ecological model of school climate (Salle et al., 2015) and Hofstede’s cultural dimensions theory (Hofstede et al., 2005), the present study used data gathered by the Organization for Economic Co-Operation and Development’s OECD (2018) Teaching and Learning International Study (TALIS) to explore the latent classes of teacher perceptions of school climate in lower-secondary schools across the U.S., Finland, and Shanghai, China. We then compared the similarities and differences in identified latent classes of teacher perceptions of climate between these three countries. Finally, we examined the predictability of three teacher background factors (gender, teaching experience in current school, and overall teaching experience) on identified latent classes. We used latent class analysis (LCA) to determine the number of latent classes of perceptions of school climate for each teacher subsample and uncover similarities and differences between the identified classes and their relationships with teacher background factors across countries. In addition, we used multigroup latent class analysis (MLCA) to examine measurement invariance across countries (Geiser et al., 2006; Ma, 2021). The following research questions and hypotheses were posed:

1. How many latent classes of teacher perceptions of school climate can be identified in lower-secondary schools in the U.S., Finland, and Shanghai, China?
(H1). Based on the cultural-ecological model of school climate (Salle et al., 2015) and previous LCA studies (Conderman et al., 2013), at least three latent classes were hypothesized for each country.
2. Are identified latent classes of teacher perceptions of school climate identical across the U.S., Finland, and Shanghai, China?
(H2). Based on the cultural-ecological model of school climate (Salle et al., 2015), identical latent classes were not expected due to the cultural sensitivity of contextual effects on teacher perceptions.
3. What are the effects of teacher background variables (i.e., teacher gender, gender, teaching experience in current school, and overall teaching experience) on the probability of being in a particular identified latent class?
(H3). According to the cultural-ecological model of school climate (Salle et al., 2015), teacher-focused school climate model (Capp et al., 2020), and empirical studies (Capp et al., 2021), teacher background factors would reflect the diversity within teachers, which was expected to produce variation in their perceptions of school climate. Specifically, female teachers are more likely to be classified into the latent class of perceiving more positive school climate than male teachers; new teachers tend to be in a latent class of perceiving less favorable climate than their experienced colleagues.

The current study is one of the first to employ a person-centered approach (i.e., LCA) to identify teacher perceptions of school climate and investigate the impact of predictors on latent class membership in a multicultural context. As previous school-climate studies have been mainly student-focused, our study could provide complementary information regarding school climate from the perspective of teachers, which has been largely overlooked in the literature (Grazia and Molinari, 2022). Teachers, who have a closer bond with students than principals and administrators, are especially likely to nurture ideas and opinions regarding school climate. Listening to teachers’ voices is vital to realizing the long-term goal of enhancing school performance and student success (Jarl et al., 2021).

In addition, applying LCA to a multicultural context may assist in better classifying teacher perceptions of school climate in different countries, which could contribute toward policy making in educational sector to promote a global, holistic, and standardize measurement on teacher wellbeing. Lastly, the research approaches and processes used in this research are potentially valuable for examining the classification of a variety of other intriguing concepts in addition to school climate (Ma, 2021).

## Materials and methods

### Sample

This study used data from the Teaching and Learning International Survey (TALIS) conducted by the Organization for Economic Co-operation and Development (OECD) between September 2017 and July 2018. Since 2008, TALIS has been conducted every 5 years, focusing on providing policy-relevant data and analysis of the key aspects of teaching and learning (OECD, 2019a). The latest cycle, conducted in 2018, aimed to investigate teachers’ learning environments and working conditions in schools (OECD, 2019b, p. 74). TALIS team used a two-stage stratified sample approach. Within the most of the participating countries and economies, 200 schools were randomly selected and invited to take part in the study, followed by drawing a random sample of 20 teachers from every selected school (Reeves and Hamilton, 2022). For a more detailed sampling procedure, please refer to the TALIS 2018 technical report (OECD, 2018).

Our study first extracted the information of 2,560 American teachers, 2,851 Finnish teachers, and 3,976 Chinese teachers in lower-secondary schools from the teacher public use file (BTGINTT3). The initial sample was filtered to exclude the observations that had any missing values in school-climate items. We run Little’s missing completely at random (MCAR) tests for each country to check for the pattern and the number of missing values (Li, 2013; Breunig, 2019). The results shown that (1) the 222 (8.67%) American cases, 306 (10.73%) Finnish cases, and 369 (9.28%) Chinese cases contains missing values and had been removed; (2) the missing patterns for the three subsamples were completely random (χ^2^ = 215.48, *df* = 216, *p* = 0.497 for the American subsample, χ^2^ = 301.98, *df* = 257, *p* = 0.056 for the Finnish subsample, and χ^2^ = 219.80, *df* = 217, *p* = 0.434 for the Chinese subsample).

Consequently, our final analytic sample included 2,338 American teachers, 2,545 Finnish teachers, and 3,607 Chinese teachers. The cluster sizes range from between 2 and 17 teachers per school for the American subsample (*M* = 7.56, SD = 2.93), between 1 and 15 teachers per school for the Finnish subsample (*M* = 7.45, SD = 2.82), and between 3 and 17 teachers per school for the Chinese subsample (*M* = 7.96, SD = 2.45). Note, although TALIS two-stage random sampling approach and our MCAR tests support the statement that our final analytical sample was drawn to be representative of the countries and region, this study cannot claim the representation of the Chinese sample as Shanghai is the largest and most developed city in China (Chen et al., 2022).

Table 1 reported the descriptive statistics of the analytical sample. Two variables were recoded: highest level of formal education (ISCED 2011 Levels < 3, 4, 5, 6, 7, 8), which we recoded into a three-choice item (ISCED 2011 Levels < 5, 6, 7–8), and employment status as a teacher at this school (ISCED 1 = Permanent employment, 2 = Fixed-term contract for a period of more than 1 school year, and 3 = Fixed-term contract for a period of 1 school year or less), which we recoded into a binary item (1 = Permanent employment, 2 = Temporary employment). All three countries had more female teachers (approximately 70%) than male teachers. For each subsample, more than 30% of teachers worked at the current school for more than 10 years, which accounted for the largest group. The distribution of overall teaching experiences (total years of working as a teacher) across three countries had a similar pattern: teachers with 0–2 years of experience accounted for the smallest proportion, whereas teachers with more than 10 years of experience comprised the largest portion. For each country, teachers aged 40–49 made up the largest proportion (30.12% for the U.S. dataset, 32.54% for the Finland dataset, and 35.83% for the China dataset). For teachers’ level education, most Finnish teachers (88.38%) pursued the highest level (ISCED 2011 Levels 7–8). Compared to American and Finnish teachers, Chinese teachers more likely treated being a teacher as their first career choice (83.91%). For employment status and teacher experiences in totally, teachers in three countries had similar experience. Generally speaking, American teachers were the most overworked (46.75 h per week) while Finnish teachers had shortest working hours (35.52 h per week). When asked whether they were supported by a mentor, 94.86% of Finnish teachers reported “No,” a much higher percentage than teachers in the other two countries. Observations with missing information were not presented as they only accounted for a small share (4.27%).

**TABLE 1:** Descriptive statistics of three teacher subsamples.

Characteristics (variable name^1^)	U.S. (*N* = 2,338)	Finland (*N* = 2,545)	Shanghai, China (*N* = 3,607)
**Gender (TT3G01)**			
Female	1,566 (66.98%)	1,759 (69.12%)	2,671 (74.06%)
Male	772 (33.02%)	786 (30.92%)	936 (25.94%)
**Teaching experience at this school (TT3G11A)**			
0–2 years	445 (19.03%)	534 (20.99%)	371 (10.29%)
3–5 years	673 (28.79%)	429 (16.86%)	542 (15.03%)
6–10 years	416 (17.79%)	531 (20.87%)	784 (21.73%)
>10 years	804 (34.39%)	1,051 (41.28%)	1,910 (52.95%)
**Teaching experience in jobs (TT3G11B)**			
0–2 years	194 (8.30%)	147 (5.76%)	231 (6.39%)
3–5 years	397 (16.98%)	315 (12.39%)	318 (8.82%)
6–10 years	358 (15.31%)	446 (17.54%)	530 (14.69%)
>10 years	1,389 (59.41%)	1,637 (64.31%)	2,528 (70.10%)
**Age (TCHAGEGR)**			
Under 25	79 (3.38%)	8 (0.33%)	110 (3.06%)
25–29	258 (11.04%)	198 (7.77%)	496 (13.63%)
30–39	670 (28.67%)	644 (25.31%)	1,199 (33.24%)
40–49	704 (30.12%)	828 (32.54%)	1,292 (35.83%)
50–59	454 (19.43%)	670 (26.33%)	495 (13.72%)
>60	172 (7.36%)	197 (7.73%)	19 (0.53%)
**Highest level of formal education (TT3G03)^2^**			
<5	6 (0.26%)	87 (3.44%)	38 (1.06%)
6	927 (39.65%)	208 (8.17%)	2,993 (82.97%)
7–8	1,405 (60.09%)	2,249 (88.38%)	576 (15.97%)
**Teaching is first career choice (TT3G08)**			
Yes	1,314 (56.20%)	1,065 (41.84%)	3,027 (83.91%)
No	1,024 (43.80%)	1,480 (58.16%)	580 (16.09%)
**Employment status as a teacher at this school (TT3G09)**			
Permanent	1,511 (64.63%)	1,657 (67.77%)	1,291 (35.80%)
Temporary	827 (35.37%)	788 (32.23%)	2,316 (64.20%)
Total working hours per week* (TT3G16)	46.75 (16.65)	35.52 (12.54)	45.51 (14.44)
Support by a mentor (TT3G21A)	366 (15.65%)	131 (5.15%)	981 (27.19%)
	1,972 (84.35%)	2,414 (94.85%)	2,626 (72.81%)

^1^Names in TALIS 2018 and TALIS Starting Strong 2018 User Guide.

^2^Highest level of formal education was measured by ISCED 2011 Levels.

## School-climate measures

Teaching and Learning International Study 2018 used 13 items in three subscales to investigate three domains of school climate: (1) teachers’ perceived disciplinary climate, (2) teacher-student relations, and (3) participation among stakeholders (OECD, 2019b, p. 332). All items shared the same question stem: “How strongly do you agree or disagree with the following statements?” Responses were rated on a four-point Likert scale (1 = strongly disagree; 4 = strongly agree). Higher values indicated better school climate. Table 2 presents the descriptive statistics for each school climate item.

**TABLE 2:** School climate items from TALIS 2018 questionnaire.

Items (variable name^1^)	U.S.	Finland	Shanghai (China)
**Teachers’ perceived disciplinary climate (TT3G41A-D)**			
When the lesson begins, I have to wait quite a long time for students to quieten down^2^	2.985 (0.812)	2.804 (0.825)	3.363 (0.657)
Students in this class take care to create a pleasant learning atmosphere	2.806 (0.758)	2.636 (0.752)	3.178 (0.616)
I lose quite a lot of time because of students interrupting the lesson^2^	2.920 (0.839)	2.851 (0.854)	3.256 (0.664)
There is much disruptive noise in this classroom^2^	2.988 (0.831)	2.808 (0.861)	3.359 (0.648)
**Teacher-student relations (TT3G49A-D)**			
Teachers and students usually get on well with each other	3.218 (0.552)	3.202 (0.486)	3.347 (0.531)
Most teachers believe that the students’ wellbeing is important	3.520 (0.556)	3.407 (0.528)	3.467 (0.531)
Most teachers are interested in what student have to say	3.248 (0.590)	3.236 (0.543)	3.339 (0.550)
If a student needs extra assistance, the school provides it	3.357 (0.597)	3.383 (0.560)	3.268 (0.567)
**Participation among stakeholders (TT3G48A-E)**			
School provides staff with opportunities to actively participate in school decisions	2.846 (0.736)	2.887 (0.649)	2.967 (0.729)
School provides parents or guardians with opportunities to actively participate in school decisions	2.849 (0.684)	2.738 (0.627)	3.022 (0.658)
School provides students with opportunities to actively participate in school decisions	2.662 (0.718)	2.871 (0.573)	2.938 (0.703)
School has a culture of shared responsibility for school issues	2.775 (0.713)	2.914 (0.605)	3.077 (0.632)
There is a collaborative school culture characterized by mutual support	2.866 (0.731)	2.939 (0.664)	3.113 (0.610)

Table displays the descriptive statistics of thirteen school-climate items in TALIS 2018 questionnaire. Item responses were rated on a four-point Likert scale (1 = strongly disagree; 4 = strongly agree).

^1^Name in TALIS 2018 and TALIS starting strong 2018 user guide; ^2^Items were reverse coded.

### Construct reliability and validity testing

We assessed the construct composite reliability (CR), McDonald’s omega, and convergent validity for each single subscale. CR is a measure of internal consistency in items (Fornell and Larcker, 1981). A CR value more than 0.7 was deemed acceptable (Nunnally and Bernstein, 1994). Like Cronbach alpha, McDonald’s omega is often used to assess reliability. However, compared to Cronbach alpha, omega requires less restrictive assumptions, and is a more accurate measure of reliability index than the Cronbach alpha (Zinbarg, 2005; Dunn et al., 2014). The coefficient omega ranges from 0 to 1, and its values greater than or equal 0.80 are popularly considered appropriate for a good reliability index of a measure (Mikkonen et al., 2022). Moreover, we conducted confirmatory factor analysis (CFA) for each subscale. A set of goodness of fit (GOF) indices was used to evaluate the mode fit, including the comparative fit index (CFI) and Tucker-Lewis index (TLI) with acceptable fit > = 0.90 (Tucker and Lewis, 1973; Hu and Bentler, 1999), and standardized root mean residual (SRMR) and root mean square error of approximation (RMSEA) with acceptable fit = < 0.08 (MacCallum et al., 1996; Hu and Bentler, 1999; Steiger, 2016). A significant factor loading of an item greater than 0.5 could be considered strongly related to the latent construct (Fornell and Larcker, 1981).

Table 3 showed eight of nine CRs exceeded the threshold value of 0.7. Teacher-student relations in Chinese subsample (0.685) was very close to the threshold value although not exceed it. All omega values were larger than the recommended value of 0.8, indicating that all domains have acceptable internal reliability. In addition, the results of CFA showed all CFIs ≥ 0.90, TLIs ≥ 0.95, RMSEAs < 0.06, and SRMRs ≤ 0.08, indicating unidimensional factor structure of the measurement model for each school climate subscale was supported with sound model fit. All individual items load strongly, which are higher than the threshold value of 0.5 and they are statistically significant. Hence, the results show good convergent validity of concept items.

**TABLE 3:** Reliability and convergent validity.

Country	School climate domains	CR	Omega	Factor loading	CFA			
					**CFI**	**TLI**	**RMSEA**	**SRMR**
U.S.	Teachers’ perceived disciplinary climate	0.867	0.912	0.514–0.714	0.996	0.992	0.033	0.007
	Teacher-student relations	0.771	0.838	0.528–0.771	0.985	0.987	0.045	0.037
	Participation among stakeholders	0.723	0.823	0.532–0.734	0.964	0.973	0.041	0.013
Finland	Teachers’ perceived disciplinary climate	0.753	0.902	0.598–0.798	0.998	0.997	0.026	0.004
	Teacher-student relations	0.784	0.842	0.619–0.752	0.957	0.951	0.043	0.021
	Participation among stakeholders	0.75	0.804	0.601–0.768	0.982	0.986	0.051	0.025
China	Teachers’ perceived disciplinary climate	0.791	0.843	0.536–0.712	0.999	0.995	0.012	0.009
	Teacher-student relations	0.685	0.901	0.531–0.801	0.981	0.928	0.045	0.034
	Participation among stakeholders	0.731	0.905	0.621–0.798	0.973	0.924	0.044	0.039

Table illustrates a summary of composite reliability (CR), McDonald’s Omega, the range of standardized factor loadings, and confirmatory factor analysis (CFA) of the measurement model for each school-climate domain. CFA was assessed by comparative fit index (CFI), Tucker-Lewis index (TLI), standardized root mean residual (SRMR), and root mean square error of approximation (RMSEA). Names in TALIS 2018 and TALIS Starting Strong 2018 User Guide. Highest level of formal education was measured by ISCED 2011 Levels.

Further, discriminant validity was assessed by comparing the square root of the average variance extracted (AVE) of a given construct and the correlations between the construct and the other constructs (Fornell and Larcker, 1981). AVE measured the amount of variance in the items explained by each subscale compared to the variance explained by measurement error (Bagozzi and Yi, 1988). An AVE of greater than 0.50 are considered acceptable (Fornell and Larcker, 1981). If the former is greater than the latter, it implies the items are more closely related to the construct than the others.

Table 4 showed all AVEs ranging from 0.521 to 0.736, exceed the suggested threshold values of 0.5. Also, all the values in the diagonal direction (bold numbers) are larger than the off-diagonal values in the corresponding rows and columns, indicating discriminant validity was satisfactory for all subconstructs in school climate. Overall, the reliability and validity assessments documented the good psychological quality of school-climate measurement.

**TABLE 4:** Discriminant validity of school-climate measures.

Country	Domains	AVE	Perceived disciplinary climate	Teacher-student relations	Participation among stakeholders
U.S.	Perceived disciplinary climate	0.714	**0.845**		
	Teacher-student relations	0.538	0.486	**0.733**	
	Participation among stakeholders	0.582	0.439	0.505	**0.763**
Finland	Perceived disciplinary climate	0.698	**0.835**		
	Teacher-student relations	0.521	0.390	**0.722**	
	Participation among stakeholders	0.531	0.545	0.465	**0.729**
China	Perceived disciplinary climate	0.736	0.858		
	Teacher-student relations	0.601	0.439	**0.775**	
	Participation among stakeholders	0.541	0.447	0.398	**0.736**

The bold values on the diagonal represent the square roots of the average variance extracted (AVE); off-diagonal values are the correlation estimates.

### Analytic process

First, to explore research question one, we conducted (single-group) LCA for each teacher subsample to identify the appropriate number of classes for teacher perceptions of school climate. Model selection was based on the following statistical criteria (Weller et al., 2020; Sinha et al., 2021): (1) Bayesian information criterion (BIC), with lower values indicating a better model-data fit, (2) Lo-Mendell-Robin Likelihood Ratio Test (LMR-LRT), with significant values indicating that the current model was better than previous one, (3) entropy, with greater values suggesting lower classification uncertainty of individuals to latent classes (Wang et al., 2017), (4) parsimony property, where a model with fewer parameters was better than a more complex model with all else being equal, and (5) theoretical interpretation, which required researchers to interpret identified classes theoretically and explain the implication for practice (Weller et al., 2020).

Second, for research question two, we assess measurement invariance across teacher samples (Lanza et al., 2007). Measurement invariance required the conditional response probabilities (within class) for school climate items equal for teacher samples in different countries. Only if this assumption holds, the identified LCA models with the same number of latent classes (if present) could be regarded as the same for different teacher subsamples; subsequently, meaningful comparisons of the class sizes could be derived (Kankaraš et al., 2018). Measurement invariance assumption was tested by comparing the model fit of fully constrained, semi-constrained, and unconstrained MLCA for the identified LCA of each teacher subsample (Parnes and Schwartz, 2022). Specifically, in a fully constrained model, both class sizes and conditional response probabilities were constrained to be the same across teacher subsamples. In contrast, the semi-constrained model allowed the class sizes to differ across subsamples, whereas the unconstrained model further allowed the variance of condition-al response probabilities across subsamples. As these three models were nested, and the distribution of the likelihood-ratio difference test was asymptotically chi-square, the model selection could be determined by comparing *G*^2, BIC, and Akaike’s Bayesian information criterion (ABIC) differences among models. A non-significant *G*^2 value and lower BIC and ABIC indicate a better model fit. If the semi-constrained model had better fitness than both fully constrained and unconstrained models, we could argue that the measurement invariance assumption was retained.

Finally, after teachers were assigned to specific school-climate categories based on their response patterns on 13 school-climate items (regardless of measurement invariance holding across countries), three teacher demographic variables were used to predict category membership: gender, teaching experience in current school, and overall teaching experience, which aims to address research question three. The specific impacts of demographic variables were interpreted for the three countries. Sampling weights were used to compensate for the disproportional selection probabilities among institutions and people and improve the generalizability of our results. We did not use multilevel modeling, as the number of participating teachers within each school (approximately 20) was too small to fulfill the sample size requirements for multilevel models (OECD, 2019a).

Latent class analysis were conducted using Mplus 8.8 (Muthén and Muthén, 2017). To assess the impact of teacher background variables (i.e., teacher gender, teaching experience in current school, and overall teaching experience) on the probability of being in a particular identified latent classes, we used the R3STEP command in Mplus 8.8 (Asparouhov and Muthén, 2014). The comparison of the models for measurement invariance used the Mplus Automation package in R software (Hallquist and Wiley, 2018). Data cleaning and other analysis procedures (e.g., CFA, McDonald’s omega) were conducted using R version 4.0.3 (R Core Team, 2020).

## Results

### Latent classes exploration (for research question one)

The optimal number of latent classes for teacher perceptions of school climate was determined by running a series of LCA for each teacher subsample, in which latent classes gradually increased from two to seven classes for the American subsample and two to five classes for the Finnish and Chinese subsamples. Table 5 details the model fit indices of all tested LCAs.

**TABLE 5:** Fit statistics for LCAs modeling teacher perceptions of school climate.

Number of profiles	AIC	BIC	ABIC	*P*-value (LMR-LRT)	Entropy
**U.S.**					
2	54709.810	55167.054	54916.053	<0.001	0.841
3	51859.498	52548.252	52170.161	<0.001	0.857
4	50358.171	51278.431	50773.264	<0.001	0.869
5	49169.372	50321.143	49688.876	<0.001	0.867
6	48384.655	49767.938	49008.581	<0.001	0.868
7	47686.943	49301.738	48415.292	0.703	0.863
**Finland**					
2	61453.725	61202.715	61453.725	<0.001	0.848
3	58942.963	58564.858	58942.963	<0.001	0.834
4	57750.203	57245.005	57750.201	<0.001	0.844
5	56954.878	56322.587	56954.878	0.5361	0.839
**Shanghai (China)**					
2	70040.610	70537.245	70286.219	<0.001	0.945
3	64894.775	65642.871	65264.742	<0.001	0.945
4	60448.050	61447.607	60942.376	<0.001	0.942
5	58941.364	60192.382	59560.050	0.743	0.948

BIC, Bayesian information criterion; ABIC, adjusted Bayesian information criterion; LMR-LRT, Lo-Mendell-Rubin likelihood ratio test, and Entropy value testing classification quality.

For the American subsample, although BIC and ABIC values decreased with the increase in latent classes, LMR-LRT indicated that a six-class model fit the model better than a five-class model (*p* < 0.001), but a seven-class model did not outperform the six-class model. However, the entropy of the six-class model (0.868) was slightly less than that of the four-class model (0.869). Considering the balance between model fitness and parsimony, the four-class model was preferable to the six-class one. In Finland and Shanghai datasets, although the five-class solutions had slightly lower AIC, BIC, and ABIC than the four-class solutions, we retained the latter as LMT-LRT was non-significant and its entropy value (almost) reached the peak (0.844 and 0.942, respectively). Therefore, our results support the hypothesis 1.

### Measurement invariance (for research question two)

After determining the optimal solution (i.e., four-class models) for each country, we compared three models (fully constrained, semi-constrained, and unconstrained models) to examine measurement invariance assumption. The results demonstrated that measurement invariance was not maintained hold across three countries, as the chi-square test between the first two models (fully constrained vs. semi-constrained) and the latter two models were both significant (Δ*G*^2 = 98.17, Δ*df* = 25, *p* < 0.01; Δ*G*^2 = 85.43, Δ*df* = 33, *p* < 0.01). We further assessed the measurement invariance assumptions for any two teacher subsamples. Again, this assumption was violated. Therefore, hypothesis 2 was supported and the following analysis conducted unconstrained models separately for each subsample.

### Naming of identified latent classes (research question two)

**FIGURE 1:**
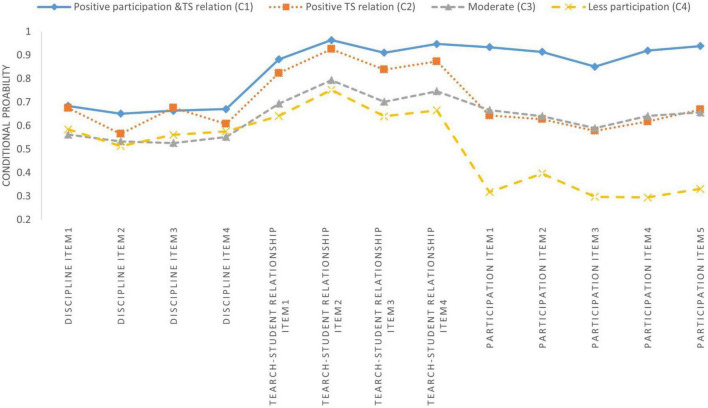
Latent classes of the U.S. subsample.

**FIGURE 2:**
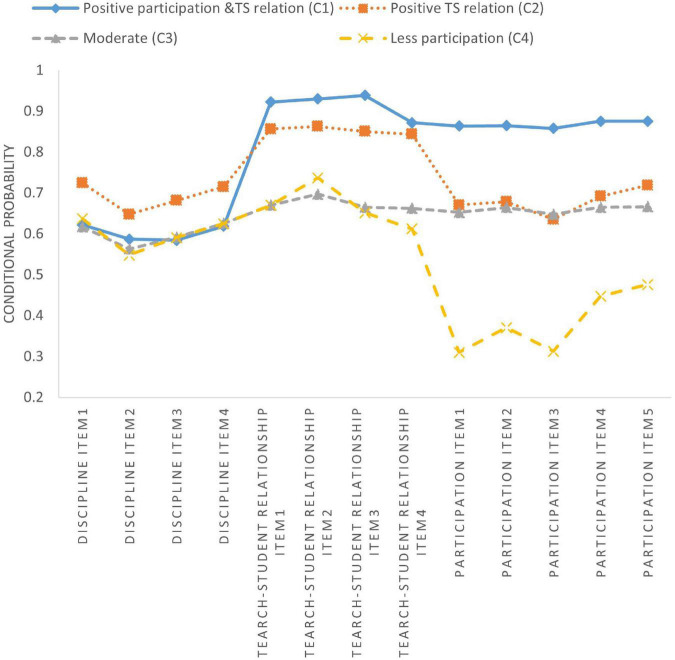
Latent classes of the China subsample.

**FIGURE 3:**
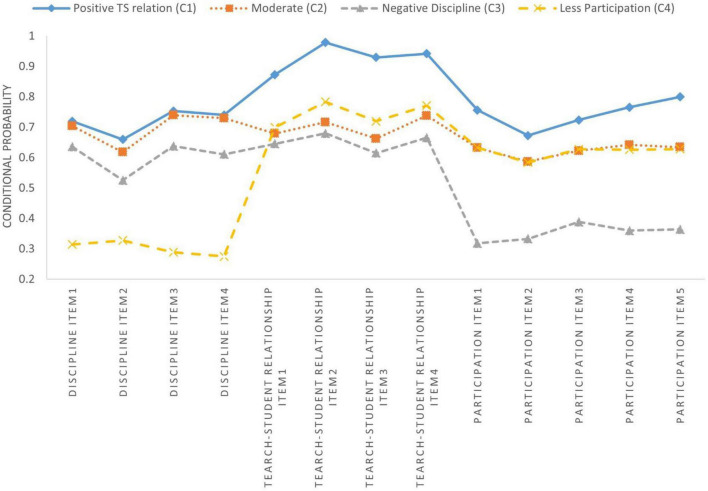
Latent classes of the Finland subsample.

Table 6 and Figures 1–3 show latent class membership and conditional probability distribution diagrams of each identified class on the 13 items for each teacher subsample.

**TABLE 6:** Latent class membership by country.

Name of latent class	U.S.	Finland	Shanghai (China)
Positive participation and TS relation	12.5% (C1)		18.5% (C1)
Positive TS relation	21.2% (C2)	27.8% (C1)	22.1% (C2)
Moderate	46.5% (C3)	34.7% (C2)	41.4% (C3)
Negative discipline		25.3% (C3)	
Low participation	22.5% (C4)	12.1% (C4)	18.1% (C4)

Table displays the latent classes and endorsement frequencies of three teacher subsamples according to teacher perceptions of school climate. TS, teacher-student relation; C1–4, Classes 1–4.

The four identified groups of American and Chinese subsamples presented similar response patterns, although the membership sizes varied (see Figures 1, 2). Class 1 reported relatively high ratings for items in the domains of participation among stakeholders and teacher-student relationships. Class 2 tended to report high perceptions of school disciplinary climate and teacher-student relationships. Class 3 comprised the largest proportion of teachers (39.5% for American data and 41.4% for Chinese data) and was characterized by moderate rates for each item. On average, teachers in class 4 had fewer opportunities to participate in school decisions. Therefore, these four classes were labeled as positive participation and teacher-student (TS) relationships, positive discipline and TS relationships, moderate, and low participation.

In contrast, based on Finnish data (Figure 3 and Table 6), Class 1 strongly agreed with items regarding teacher-student relationships and responded moderately (i.e., agree or disagree) to items in the other two domains. Thus, they were labeled as the positive TS relationships category. Class 2, labeled as moderate, tended to report a moderate attitude on each item. Classes 3 and 4 had a negative experience with disciplinary climate and participation among stakeholders, respectively. Thus, class 3 was labeled as negative discipline, and class 4 as low participation.

### Predictors of latent class membership (for research question three)

Table 7 reports the results of multinomial logistic regression using teacher gender and teaching experience as predictors of class membership. The most positive class was used as the reference class, meaning positive participation and TS relationships for the American and Chinese subsamples, and positive TS relationships for the Finish subsample.

**TABLE 7:** Multinomial logistic regression results (compared to most positive class).

	Positive participation and TS relation		Positive TS relation		Negative discipline		Low participation	
	**β (SE)**	**OR**	**β (SE)**	**OR**	**β (SE)**	**OR**	**β (SE)**	**OR**
**U.S.**								
Gender^1^	−0.034 (0.234)	0.966	−0.081 (0.262)	0.922			−0.544* **(0.228)**	**0.580**
**Experience at school^2^**								
0–2 years	0.048 (0.349)	1.049	0.013 (0.419)	1.013			0.383 (0.397)	1.467
3–5 years	0.344 (0.368)	1.411	−0.063 (0.401)	0.939			0.434 (0.358)	1.543
6–10 years	0.350 (0.285)	1.419	0.323 (0.364)	1.381			0.887** **(0.294)**	**2.428**
**Experience in job^2^**								
0–2 years	0.822 (0.524)	2.275	0.881 (0.483)	2.412			1.277* **(0.545)**	**3.585**
3–5 years	−0.008 (0.426)	0.992	0.726 (0.470)	2.067			0.534 (0.458)	1.706
6–10 years	0.468 (0.377)	1.597	0.263 (0.357)	1.301			0.458 (0.349)	1.580
**Finland**								
Gender			−0.055 (0.174)	0.947	0.171 (0.138)	1.186	0.152 (0.128)	1.164
**Experience at school**								
0–2 years			0.338 (0.285)	1.402	−0.088 (0.218)	0.916	−0.028 (0.195)	0.972
3–5 years			0.367 (0.271)	1.443	−0.268 (0.212)	0.765	−0.362 (0.199)	0.697
6–10 years			0.226 (0.226)	1.254	−0.233 (0.190)	0.792	−0.181 (0.169)	0.834
**Experience in jobs**								
0–2 years			0.350 (0.422)	1.419	0.819** **(0.320)**	**2.267**	0.171 (0.309)	1.186
3–5 years			−0.168 (0.332)	0.846	0.794*** **(0.240)**	**2.213**	0.320 (0.234)	1.378
6–10 years			−0.278 (0.272)	0.757	0.978*** **(0.194)**	**2.659**	0.391* **(0.187)**	**1.478**
**Shanghai (China)**								
Gender	−0.217 (0.113)	0.805	−0.121 (0.109)	0.886			−0.002 (0.109)	0.998
**Experience at school**								
0–2 years	−0.192 (0.265)	0.825	0.568* **(0.227)**	**1.765**			−0.212 (0.232)	0.809
3–5 years	−0.159 (0.192)	0.853	0.212 (0.197)	1.236			0.047 (0.177)	1.048
6–10 years	0.011 (0.170)	1.011	0.454** **(0.147)**	**1.575**			0.126 (0.149)	1.135
**Experience in jobs**								
0–2 years	0.169 (0.317)	1.184	−0.124 (0.279)	0.884			0.670* **(0.267)**	**1.954**
3–5 years	0.145 (0.237)	1.156	−0.140 (0.242)	0.869			0.587** **(0.205)**	**1.799**
6–10 years	−0.071 (0.192)	0.932	−0.204 (0.167)	0.815			0.267 (0.165)	1.306

Table reports the results of multinomial logistic regression using teacher gender and teaching experience as predictors of latent class membership. The most positive class was used as the reference class, meaning positive participation and TS (teacher-student) relationships for the American and Chinese subsamples, and positive TS relationships for the Finish subsample. Bold estimates indicate significant differences. *p < 0.05, **p < 0.01, ***p < 0.001. Reference groups for gender^1^ and teaching experience at school/in jobs^2^ are male and >10 years, respectively.

For the American data, female teachers had 42.0% (OR = 0.580) lower odds of being in the low participation class than male teachers. Teachers working in the current school for 6–10 years were 2.428 times more likely to be in the low participation class than teachers working in the current school for more than 10 years. Moreover, the odds of being in the low participation class compared with positive participation and TS relationships was 1.277 times higher for teachers with 0–2 years of teaching experience.

The results for the Finnish subsample revealed that teachers with less than 10 years of teaching experience were more likely to be in the low participation class (OR = 1.478) or negative discipline class (OR = 2.267, 2.213, 2.659 for teachers with 0–2, 3–5, 6–10 years of experience, respectively).

In the Chinese sample, the odds of being in the moderate class were 0.568 and 0.454 times higher for teachers working in the same school for 0–2 and 6–10 years, respectively. The odds that teachers with 0–2 and 3–5 years of experience would experience low participation were 1.954 and 1.799 times higher, respectively, than those of teachers with more than 10 years of experience.

In sum, for teacher gender, only the American subsample supports hypothesis 3 that assumes female teachers had a more positive feeling of school climate than male teachers. For teaching experience, although the patterns are not the same across three countries, all significant coefficients of teaching experience were positive. This means teachers in all three countries with less than 10 years of experience were more likely to be in the less positive class than those with more than 10 years; thus, hypothesis 3 about teaching experience was supported.

## Discussion

The impact of school climate on student and teacher outcomes has received increasing attention (Konishi et al., 2022; Yang et al., 2022; Zacharia and Yablon, 2022; Finch et al., 2023, p. 20). However, most previous studies have focused on students’ perceptions of school climate and ignored teachers’ perceptions (Wang and Degol, 2016; Gonzálvez et al., 2022; Grazia and Molinari, 2022). Furthermore, most studies on school climate and its predictors were conducted in a single context (Oder and Eisenschmidt, 2018; Sanchez et al., 2020; Marchante et al., 2022), lacking the exploration of cultural and contextual differences.

This study represents one of the first attempts to investigate the cross-cultural differences in teacher perceptions of school climate. We explored the latent classes of teacher perceptions of climate in lower secondary schools in the U.S., Finland, and Shanghai, China (Table 5) and the impact of predictors on latent class memberships. We found that: (1) A four-class solution was determined to be the optimal model for each teacher subsample; (2) Three teacher subsamples presented extremely heterogenous response patterns for 13 school climate items, and identified four classes of each teacher subsample as quantitatively different from each other (Figures 1–3 and Table 6); (3) The impacts of three predictors (gender, teaching experience at current school, and overall teaching experience) on teacher classification varied significantly across countries (Table 7).

The identified latent class differences in teacher perceptions of school climate and predictor differences of latent class membership indicated that culture played a critical role in impacting teacher perceptions of school climate (Gonzálvez et al., 2022), which aligns with the cultural-ecological model of school climate (Salle et al., 2015). Although the cross-country disparities in how students and parents perceive school climate have been documented in the literature (Yang et al., 2013, 2021; Larson et al., 2020), this cross-cultural study advanced the understanding of differences in school climate across countries and could inform the design and development of school climate initiatives and programs from teacher perspectives.

### Latent classes comparison

For research question one, LCAs identified four classes for each country (Figures 1–3 and Tables 5–6), supporting hypothesis 1. School climate, which is a complex concept, cannot be simply dichotomized as positive or negative. The four classes identified for each subsample were crucial, as they presented a comprehensive description of the ways in which teachers perceived school climate differently (Rosato and Baer, 2012; Capp et al., 2021; Herman et al., 2023). Three universal classes were identified across three countries: positive TS relationships (high rates of teacher-student relationships), moderate (moderate rates of each domain of school climate), and low participation (low participation rates among stakeholders) class. The three countries had similar proportions of teachers in the positive TS relationship class (U.S., 21.2%; Finland, 25.8%; China, 22.1%). Unsurprisingly, teachers in the moderate class accounted for the largest groups in each country (U.S., 46.5%; Finland, 38.1%; China, 41.4%). It would be meaningful and informative to promote this class to shift toward other classes with more positive views on school climate.

For the low participation class (few opportunities to participate in school decisions), only 12.1% of Finnish teachers were assigned to this class, and 18.1% of Chinese teachers; however, the U.S. indicated a nearly double rate (22.5%). As documented by previous studies (Elo and Nygren-Landgärds, 2021), compared to Chinese and American counterparts, Finnish teachers reported higher rates of autonomy and decision-making power on school policies and management (Xia et al., 2017; Hemphill, 2018). Consequently, they are more active in school management and engagement. In contrast, college entrance examinations is the core concern of Chinese primary and secondary education systems as it is almost the only one avenue for most students to change their fate (Lo, 2019; He et al., 2022). Therefore, teaching for Chinese teachers are heavily textbook-dependent and primarily exam-focused so that teachers lose their autonomy and become subservient (Day, 2019). Different from Finland and China, performance-based accountability policies (e.g., No Child Left Behind, Every Student Succeed) strongly shape and frame at all levels of American education system (Mehta, 2014; Maaranen and Afdal, 2022). Under these policies, each state and school district require teachers to mandate the implementation of particular standards for classroom teaching and management, which have been undermining teacher autonomy (Maaranen and Afdal, 2022; Levatino et al., 2023).

In addition to moderate and low participation classes, one unique class was identified for each country. American and Chinese datasets disclosed a positive participation and TS relationship class (i.e., high rates of participation among stakeholders and positive teacher-student relationships); however, the Finnish subsample consisted of a negative discipline class (low level of disciplinary climate). The different attitudes toward student academic performance and class management across countries may help explain the disparities. Teachers in Finland were far less worried about the grade level of their students, the quality of assigned homework, and standardized tests than their counterparts in the U.S. and China. Consequently, students in Finnish schools have greater freedom for introspection and experimentation (Partanen, 2011). However, this may come at the price of school discipline and regulations. As such, 23.4% of all Finnish educators were considered to experience poor school discipline. In contrast, under the high stresses of college entrance examination and accountability policies for Chinese and American teachers, respectively, they is more likely to maintain positive classroom discipline as it often benefits students their academic performance (Rodriguez and Welsh, 2022).

Finally, in terms of the positive TS relationship class and more inclusive classes (i.e., positive participation and TS relationship class), 40.6% of Chinese teachers were assigned to these classes, which was significantly higher than American (33.7%) and Finnish (27.8%) teachers (Table 4). Compared to their counterparts in the U.S. and Finland, Chinese students have a more optimistic perspective of the teacher-student connection (Yang et al., 2013). A high degree of respect for teachers is valued in China due to Confucian virtue, which guides children to strive for perfection (including self-discipline) and honor their parents (Hui et al., 2011). Chinese students’ commitment to studying, desire for self-improvement, and respect for their teachers are likely major contributors to the country’s high levels of student-teacher attachment, low rates of disruptive behavior, and impressive academic achievements. Undoubtedly, many students in the U.S. and Finland have these traits. However, American and Finnish student populations, particularly those beyond the primary school level, are less likely to exhibit these traits than their Chinese counterparts (Culpeper et al., 2010; Qu and Pomerantz, 2015; Qu et al., 2016). Moreover, compared to American and Finnish teachers, Chinese teachers tend to use praise and reward prosocial behaviors (Teddlie and Liu, 2008; Bear et al., 2016). In sum, Confucian values and student behavior management in Chinese schools help promote positive teacher-student relationships.

### Measurement non-invariance

Research question two aimed to explore whether the identified four latent classes of teacher perceptions of school climate for each country are identical. As the four latent classes of American and Chinese subsamples were named identically and that for Finnish subsample was different, it was obvious that the identified latent classes across three countries were different, supporting hypothesis 2. In addition, caution should be taken regarding the present findings, as measurement invariance was violated. Theoretically, identified classes were not directly comparable across countries, supporting the idea of school climate being a culturally and contextually sensitive construct (Salle et al., 2015; Chen et al., 2020; Del Toro and Wang, 2021; Yang et al., 2021; Na’imah et al., 2022). Making meaningful cross-cultural comparisons requires avoiding three types of biases: construct, method, and item (Schmidt et al., 2020). Construct bias occurs when teachers from different countries have different understandings of definitions, concept-related aspects, or behaviors. Currently, we cannot exclude the possibility that the conceptualization of school climate for teachers across countries was different (Salle et al., 2015).

Method bias indicates that nuisance arises due to inappropriate sampling, instrument design, and administration process. One example of administration bias is that miscommunication is almost certain to occur between testers and testees from different cultural backgrounds (van de Vijver, 2002; Quesque et al., 2022). Item bias is possible when an item has different meanings across cultures (Benítez et al., 2022). To avoid item bias, valid and reliable measures are recommended. However, the significant differences in response patterns on school climate items across countries in the present study indicated that item bias might occur and a more valid and reliable tool for assessing teacher perceptions of school climate is necessary to explore cross-cultural variation.

### Association of latent class and teacher background

Regarding the impact of predictors on latent classes of teacher perceptions of school climate, varied patterns of cross-cultural differences were found. Our results partly supported hypothesis 3.

#### Teacher gender

As shown in Table 5, only female teachers in the U.S. had a lower probability of being categorized into the low participation class. This partly validated the findings of previous studies (Drugli, 2013; Capp et al., 2021), which indicated that female teachers connected with students with higher levels of affinities and less conflict, contributing to positive teacher-student relationships and school climate. However, this was not the case in the Finnish and Chinese subsamples. The present data did not reveal reasons for this disparity; however, they highlighted diverse patterns of experiences for female and male teachers across countries and indicated the importance of exploring cultural differences (Salle, 2018; Enkhtur et al., 2022).

#### Years of teaching

As shown in Table 5, although the patterns are not the same in different countries, all significant coefficients of teaching experience were positive, indicating that teachers in all countries with less than 10 years of experience were more likely to be in the less positive class than those with more than 10 years of experience. Previous studies have suggested that less experienced teachers are often busy just attempting to survive under the disadvantages of less collegial support and decision-making authority (Murray-Orr and Mitton-Kukner, 2017; Thomas et al., 2021). Thus, they are more likely to experience emotional weariness (Grayson and Alvarez, 2008) and quit their jobs (Djonko-Moore, 2016). Those combinations of stressors might account for a more unpleasant climate experience (Capp et al., 2021). However, our research cannot explain why years of teaching experience impact different groups in three countries, which is a gap for future research.

### Limitations and future research

Although the current study is one of the first to investigate cross-cultural differences and similarities in teacher perceptions of school climate, it has several limitations. First, TALIS 2018 used 13 items to measure three domains of school climate. Thus, it may not comprehensively reflect teachers’ perceived school climate, as a systematic review revealed that school climate consists of four domains: school safety, academic climate, community, and institutional environment (Wang and Degol, 2016). Further research could design a more comprehensive scale to measure teacher perceptions of school climate. Second, due to the cross-section nature of the TALIS data, it was not possible to determine causal links between the perceptions of school climate and teacher gender and teaching experience in this study although it does not rule out the potential. Future research with experimental or longitudinal designs is required. Third, the results of this study were inferred from an examination of the U.S., Finland, and Shanghai, China. It may not be applicable to other countries and economies.

Fourth, although self-reported measures are beneficial for evaluating teachers’ subjective opinions of the school climate, it may be impacted by social desirability bias (Fisher and Katz, 2000), which is particularly pronounced in the Chinese sample. For instance, due to the cultural difference, Chinese respondents are more likely to choose moderate and extremely positive responses compared to their American and Finnish counterparts (Harzing, 2006; Kjærnsli and Lie, 2011), which could threaten our conclusions. Finally, to more holistically assess the disparities in how schools are seen and perceived by their communities throughout the world, researchers may use multi-method techniques to examine school climate from multi-stakeholders (e.g., students, parents, and principals) in multicultural contexts (Yang et al., 2021).

### Practical implications

In the contemporary context of growing international educational initiatives and programs, the present findings have several significant implications for measuring, comprehending, and comparing school climate internationally and building a healthy school climate in various countries. First, this study reveals that LCA is a useful method for classifying the teacher perceptions of school climate (or various other constructs of interest). Using arbitrary threshold values or the more conventional cluster analysis may not provide as rigorous or trustworthy classification findings as the data-driven LCA (DiStefano and Kamphaus, 2006; Loades et al., 2022).

Second, our results suggest that more than half of teachers were classified in the moderate or less desired (i.e., low participation, negative discipline) class. Thus, tailored strategies and programs are necessary to foster a positive school climate. As teacher perceptions of school climate are entangled with student behaviors (e.g., teacher-student relationships, school discipline and order, bullying) (O’Brennan et al., 2014), school climate interventions must simultaneously consider teacher and student reactions. Teachers always play a critical role in successfully implementing student outcome interventions (e.g., anti-bullying intervention) (Fischer and Bilz, 2019), which could foster not only student perceptions of school climate but also those of teachers (Acosta et al., 2019). The process of improving the school climate for teachers may influence the kinds of interventions that are necessary to support students. That is, if teachers sense a gradually improving school climate, it is quite likely that the experiences of the students and their outcomes will also improve (Capp et al., 2021).

Third, this study revealed that the TALIS 2018 school climate measure might not be psychometrically sound in terms of measurement invariance. Previous studies have indicated this kind of measurement problem is very common for international assessments (Schleicher, 2019). The Program of International Student Assessment, a study investigating the performance of 15-year-old students from more than 75 countries, presented differential item functioning when analyzing various language versions of the same scales (Hopfenbeck et al., 2018). In addition, it has been shown that PISA scales are more comparable across countries in the Western hemisphere than they are across countries in the Middle East or Asia due to the linguistic and cultural variations between those regions (Grisay et al., 2007; Khan et al., 2022). Although without direct evidence for TALIS scales, we believe TALIS scales may meet similar challenges as both of them are released under the supervision of OECD. Thus, it is necessary to consider cultural factors and design a more reliable and valid scale for cross-country comparison.

Finally, consistent with the cultural-ecological model of school climate (Salle et al., 2015), the current results emphasize that the variant impacts of several predictors (i.e., gender, teaching experience) on school climate classes across the countries, indicating educators should consider cultural differences when drawing on the experiences from other countries (Ma, 2021; Wang et al., 2022). For example, our results found that female teachers in the U.S. are more likely to feel positive school climate than male teachers, while it is not the case for Finnish and Chinese teachers. Therefore, compared to Finland and China, educators and policy-makers in the U.S. need to consider more gender differences in teacher perceptions of school climate when designing prevention and intervention programs.

## Data availability statement

The datasets presented in this study can be found in online repositories. The names of the repository/repositories and accession number(s) can be found below: https://www.oecd.org/pisa/data/2018database/.

## Ethics statement

Ethical review and approval was not required for the study on human participants in accordance with the local legislation and institutional requirements. Written informed consent from the patients/participants or patients/participants legal guardian/next of kin was not required to participate in this study in accordance with the national legislation and the institutional requirements.

## Author contributions

RJ developed the ideas. MZ and RJ wrote the manuscript. Both authors contributed to the article and approved the submitted version.
